# On the Quenching of Electron Temperature in Inductively Coupled Plasma

**DOI:** 10.3390/ma16083219

**Published:** 2023-04-19

**Authors:** Inho Seong, Si-jun Kim, Youngseok Lee, Chulhee Cho, Wonnyoung Jeong, Yebin You, Minsu Choi, Byeongyeop Choi, Shinjae You

**Affiliations:** 1 Applied Physics Lab for PLasma Engineering (APPLE), Department of Physics, Chungnam National University, Daejeon 34134, Republic of Korea; 2 Institute of Quantum Systems (IQS), Chungnam National University, Daejeon 34134, Republic of Korea

**Keywords:** plasma diagnostics, electron temperature, electron temperature quenching, skin effect

## Abstract

Electron temperature has attracted great attention in plasma processing, as it dominates the production of chemical species and energetic ions that impact the processing. Despite having been studied for several decades, the mechanism behind the quenching of electron temperature with increasing discharge power has not been fully understood. In this work, we investigated the quenching of electron temperature in an inductively coupled plasma source using Langmuir probe diagnostics, and suggested a quenching mechanism based on the skin effect of electromagnetic waves within local- and non-local kinetic regimes. This finding provides insight into the quenching mechanism and has implications for controlling electron temperature, thereby enabling efficient plasma material processing.

## 1. Introduction

Plasma processing has been widely used in material processing, as it generates radicals that participate in chemical reactions on the material surface [1]. Plasma also produces energetic ions that carry kinetic energy to the surface, causing physical sputtering and enhancing chemical reactions on the material surface [2,3]. Since the processing mechanism is governed by both chemical reactions and energetic ion impacts, the production of radicals and ions is significant in material processing [4,5,6].

Since electrons in bulk plasma play a substantial role in the production of radicals and ions, understanding their behavior in plasma is crucial [5]. The electrons are characterized by the electron energy distribution function (EEDF), which gives the electron density ($n_{e}$) and electron temperature (T_e_) and involves information about their kinetic dynamics [7,8]. The $n_{e}$ is highly related to processing rates in plasma processing such as etching [4], deposition [9], and ashing rates [6], since the ion flux is proportional to the electron density. This fact leads to the use of the $n_{e}$ as a critical parameter in next-generation process monitoring technology [10,11,12,13,14]. Furthermore, the $n_{e}$ and the T_e_ are related to ion- and radical production since the particle production mechanism in processing plasma is dominated by electron impact collisions such as electron impact dissociation, ionization, and excitation. The electron impact dissociation collision produces radical species, which have a huge chemical reactivity and react with the material surfaces and other radicals on the surfaces. Electron-impact ionization collision produces ion species, which strike the material surfaces and activate the surfaces by transferring their kinetic energy to it [5]. Electron-impact excitation produces metastables, which produce other radicals [6] and ions [15] or thermal energy on the material surfaces. In terms of particle production, the T_e_ has a greater influence on production than the $n_{e}$ because creation reactions have a non-linear growth rate with T_e_ [16,17], as described by the Arrhenius equation. Therefore, T_e_ is a significant factor for optimizing material processing and its application.

Various studies have investigated the behavior of T_e_ in an inductively coupled plasma source, which is a promising tool for material processing [18]. At high electron density and low-pressure environments (<a few mTorr), an increase in T_e_ with discharge power has been reported [19]. This increase is due to the ’Maxwellization’ of low-energy electrons through electron–electron (e-e) collisions, in which they gain energy from high-energy electrons via e-e collisions. They finally reach a thermal equilibrium state in which their velocity distribution follows a Maxwellian distribution. At higher pressure environments (>10 mTorr), T_e_ can also increase through neutral gas heating, provided that the electron–neutral (e-n) collision rate is larger than the e-e collision rate. The reduction of gas density in the bulk plasma due to neutral gas heating causes inefficient ionization, leading to an increase in T_e_ [20].

On the other hand, quenching of T_e_ has been observed at high electron density and high pressure [15,19], where e-n collisions are more frequent than e-e collisions. This quenching has been explained by multi-step ionization [15,19,21]. The ionization of excited species is called multi-step ionization and that of grounded species is called single-step ionization. When the contribution of the multi-step ionization to total ionization is greater than that of single-step ionization, T${}_{e}$ decreases due to enhanced ionization efficiency; the excited species has a lower ionization threshold energy than the grounded species. However, the impact of the multi-step ionization on T${}_{e}$ quenching was found to be smaller than the experimental result and it therefore has yet to be fully understood. In this study, we observed T${}_{e}$ quenching in a relatively low-density plasma environment compared to [15] and investigated it using another analysis scheme called the skin effect of electromagnetic waves.

This paper is organized as follows: in the next section, we describe the experimental apparatus and setup. In Section 3, we analyze the T${}_{e}$ quenching through measurements of the electron energy probability function, and suggest its mechanism in detail. In the final section, we summarize our findings.

## 2. Experiment Setup

**Figure 1:**
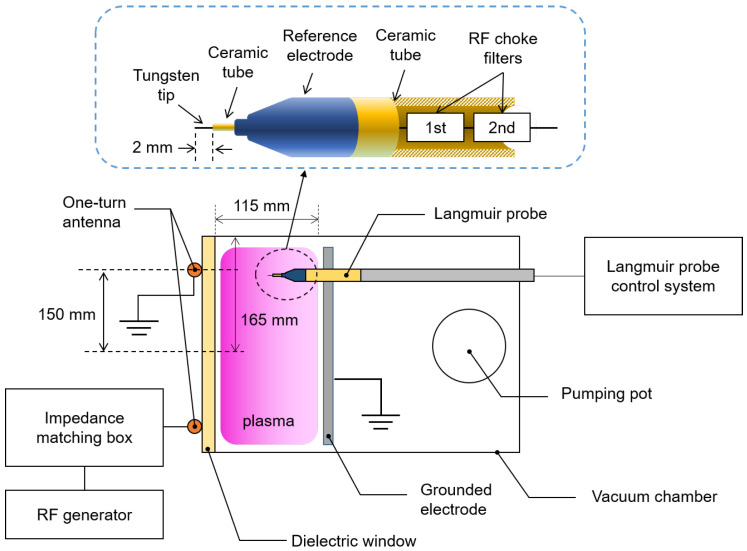
Schematic diagram of experiment setup involving an inductively coupled plasma source and Langmuir probe.

Figure 1 shows a schematic diagram of the experimental setup. Argon gas (99.999% purity) of 2 standard cubic centimeters per minute (sccm) was injected into a cylindrical stainless steel vacuum chamber with a radius of 165 mm through a mass flow controller (MFC, LineTech Inc., Deajeon, Republic of Korea). A rotary pump (DS102, Agilent Inc., Santa Clara, CA, USA) drew Argon gas through the pumping port to sustain the chamber pressure. The pressure was regulated by adjusting the open/close ratio of a gate valve. Discharge power from a 13.56 MHz power generator (YSR-06MF, YongSin RF Inc., Hanam-si, Republic of Korea) was applied to a one-turn copper antenna via an RF impedance matcher (YongSin RF Matcher, YongSin RF Inc., Hanam-si, Republic of Korea), which played a role in the impedance matching between the load impedance and the power generator. The 13.56 MHz power applied to one-turn antenna inductively couples with seed electrons inside the chamber through the dielectric window (Al_2_O_3_) and then plasma forms.

We used a Langmuir probe, a precise diagnostic instrument, to measure electron parameters [5]. As shown in Figure 1, the Langmuir probe was inserted into the vacuum chamber through the grounded electrode. The tungsten tip had a diameter of 0.15 mm and a plasma-exposed length of 2.0 mm. For radio-frequency (RF) compensation, we used in-house RF chokes for the first and second harmonics to block the RF voltage drop on the sensing resistor [22]. Furthermore, to reduce the probe tip impedance, we also used an in-house reference electrode with an aluminum-anodized surface. We employed a commercial controller (WP SLP Controller, P&A Solutions, Seongdong-gu, Seoul, Republic of Korea) to sweep voltages (V) and measure current (I) of the Langmuir probe.

The electron energy probability function ($g_{\mathrm{EEPF}}\left(E\right)$) is derived from the second derivatives of the measured V–I curve. Then, the $n_{e}$ and effective electron temperature (T${}_{\mathrm{eff}}$) can be determined as

(1) $$n_{e}=\int_{0}^{\infty }g_{\mathrm{EEPF}}\left(E\right)dE$$

and

(2) $$T_{\mathrm{eff}}=\frac{2}{3}\frac{1}{n_{e}}\int_{0}^{\infty }Eg_{\mathrm{EEPF}}\left(E\right)dE.$$

Here, we used the term T${}_{e}$ as the T${}_{\mathrm{eff}}$ for simplicity.

## 3. Results and Discussion

**Figure 2:**
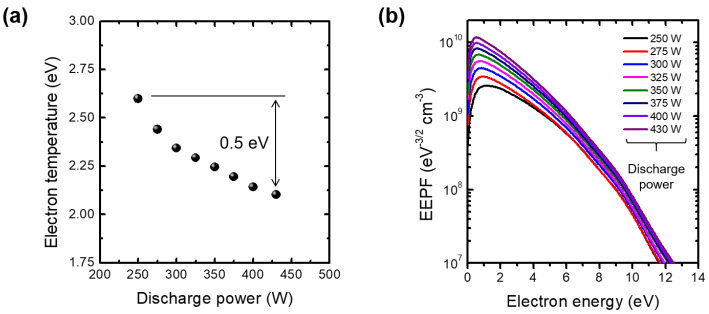
(**a**) Measured electron temperature (T${}_{e}$) over the discharge power and (**b**) electron energy probability function (EEPF) with various discharge powers at pressure of 206 mTorr. The Langmuir probe position is 58 mm, which is the center between the dielectric window (Al_2_O_3_) and the grounded electrode.

Figure 2a,b show the measured T${}_{e}$ and the EEPF, respectively, at the center between the dielectric window and the grounded electrode at 206 mTorr. A large amount of quenching of the T${}_{e}$ was observed up to 0.5 eV. Previously, similar quenching scales (≈0.4 eV) at 200 mTorr with 6.78 MHz RF power and lower scale (≈0.1 eV) at 50 mTorr with 13.56 MHz RF power [20] were reported. In both [19] and [20] it is noted that previous T${}_{e}$ measurements were conducted at $n_{e}$, several orders of magnitude larger than those in this work. Furthermore, the theoretical estimation of the multi-step ionization effect on T${}_{e}$ quenching was below the 0.1 eV order [15], which is lower than the measurement result in Figure 2a. Hence, the quenching measured in this work implies the existence of another quenching mechanism.

We found that the skin effect of electromagnetic waves can explain the T${}_{e}$ quenching. Here, the skin effect refers to the transformation of incident electromagnetic waves to evanescent waves in plasma [5,23]. Details are presented in the next paragraph, and here we focus on the validity of the skin effect analysis in this environment. To analyze the skin effect on T${}_{e}$, it is significant to investigate the electron kinetic regime, namely, whether it is under local- or non-local kinetic regimes [5]. In the local kinetic regime, the electric field in plasma heats electrons near the field [23]. Here, the electron heating means the energy gain process of electrons through the electric field and can determine the electron temperature. To figure out the local kinetic regime in this system, comparing electron energy relaxation length ($\lambda_{E}$) with chamber scale and measuring electron density profile are important [7,8].

**Figure 3:**
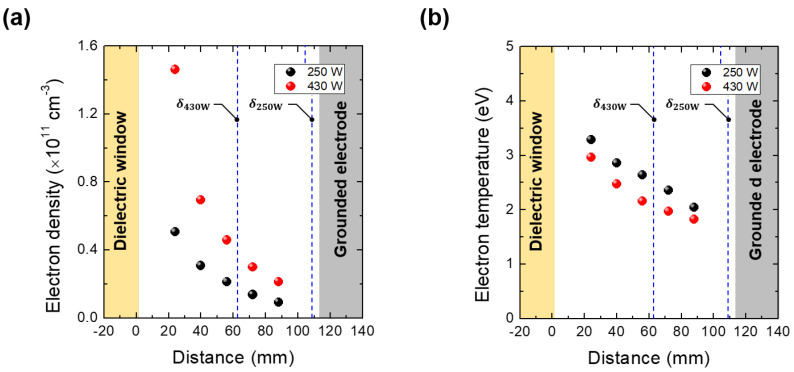
(**a**) Electron density and (**b**) temperature profiles with discharge powers of 250 W and 430 W at 206 mTorr. The average skin depths ($\delta_{250W}$ of 108.9 ± 14.6 mm and $\delta_{430W}$ of 60.6 ± 8.4 mm) are marked as the dashed lines.

The $\lambda_{E}$ is defined as [23]

(3) $$\lambda_{E}=\lambda_{\mathrm{el}}{\left[\frac{2m_{e}}{M}+\frac{\nu_{\mathrm{ee}}}{\nu_{m}}+\frac{2eE_{\mathrm{exc}}\nu_{\mathrm{exc}}}{3k_{B}T_{e}\nu_{m}}+\frac{2eE_{\mathrm{iz}}\nu_{\mathrm{iz}}}{3k_{B}T_{e}\nu_{m}}+3\frac{\nu_{\mathrm{iz}}}{\nu_{m}}\right]}^{-1/2},$$

where $\lambda_{\mathrm{el}}$ is the mean free path for e-n collisions, *e* is elementary charge, $m_{e}$ is the electron mass, *M* is the ion mass, $\nu_{\mathrm{ee}}$ is the e-e collision frequency, $\nu_{m}$ is the e-n collision frequency, $E_{\mathrm{exc}}$ and $\nu_{\mathrm{exc}}$ are the excitation threshold energy and its collision frequency, respectively, and $E_{\mathrm{iz}}$ and $\nu_{\mathrm{iz}}$ are the ionization threshold energy and its collision frequency, respectively. Here, we calculated the first and second terms (elastic and e-e collisions) from zero to 11.55 eV, which is the threshold energy of excitation collision and we considered all terms in the case of high electron energy larger than 11.55 eV. The exact forms of all parameters are described in Appendix A. At a pressure of 206 mTorr, the estimated $\lambda_{E}$ (≈10 mm) within the range from 2 eV to 11.55 eV, where electrons effectively gain energy through Ohmic heating, is much smaller than the chamber length. Furthermore, the electron density profile leans toward the high electric field region in the local kinetic regime. Figure 3a shows the measured electron density profile, which leans toward the dielectric window. Since the one-turn antenna is positioned behind the dielectric window, the electric field increases as it gets closer to the window. Considering two factors, $\lambda_{E}$ and density profile, this pressure regime belongs to the local kinetic regime. Hence, the electric field at the centre plays a significant role in electron heating to analyze the T${}_{e}$ quenching shown in Figure 2a.

As we proved, the pressure condition of 206 mTorr belongs to the local kinetic regime. The local electric field is then crucial. In an inductively coupled plasma source, a time-varying electric field is induced by a time-varying magnetic field inside a vacuum chamber. It propagates into plasma as an electromagnetic (EM) wave. The incident EM wave becomes an evanescent wave in bulk plasma with a decay constant, called skin depth (δ), which is a characteristic penetration depth of electromagnetic waves in a conductive medium. The B-dot probe is a precise measurement tool for electric field measurement in inductively coupled plasma [24]. Since measuring magnitudes of electric fields in three dimension axes ($E_{x}$, $E_{y}$, and $E_{z}$) requires complicated probe structure, we estimated the δ by measuring plasma parameters for simple analysis.

The δ is the inverse of the imaginary part of the wavenumber ($k_{i}$) of EM wave in plasma and is defined as

(4) δ=ki−1=Imωcϵp(ω)−1

where ω is the EM wave angular frequency, *c* is the speed of light, and $\epsilon_{p}\left(\omega \right)$ is the dielectric constant of plasma defined as [5]

(5) $$\epsilon_{p}\left(\omega \right)=\epsilon_{0}\left(1-\frac{\omega_{\mathrm{pe}}^{2}}{\omega \left(\omega -i\nu_{m}\right)}\right)$$

where $\epsilon_{0}$ is the permittivity in vacuum and $\omega_{\mathrm{pe}}$ is the angular plasma frequency defined as

(6) $$\omega_{\mathrm{pe}}=\sqrt{\frac{e^{2}n_{e}}{\epsilon_{0}m_{e}}}$$

**Figure 4:**
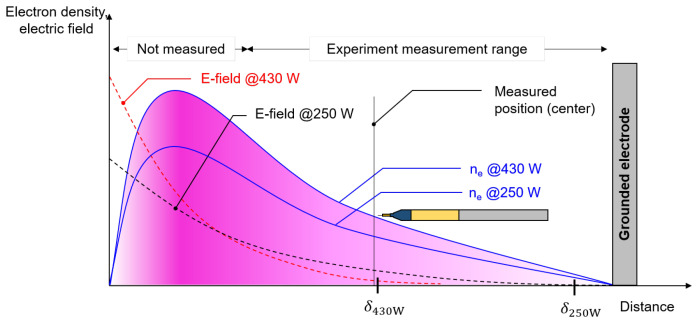
Schematic diagram of electron density and electric field profiles at different discharge powers.

As the EM wave frequency is fixed at 13.56 MHz, the skin effect depends on the $n_{e}$ and the $T_{e}$. Figure 3a,b show the measured $n_{e}$ and $T_{e}$ distributions. The result shows that both parameters have density gradients. Skin depth under inhomogeneous plasma conditions differs from that in Equation (4) [25,26]. Here we used the average skin depth, which is the average value of skin depths with $n_{e}$ and $T_{e}$ at each measurement position for simplicity rather than using the complex form of skin depth in [26]. Comparing average skin depth is reasonable since the density profile shapes at 250 W and 430 W are similar (Figure 3a) and, thus, shape is not a crucial factor for comparing their δs. The average skin depth is marked as the dashed line in Figure 3. As the discharge power increases, the δ decreases from 108.9 ± 14.6 mm at 250 W to 60.6 ± 8.4 mm at 430 W. It means that the electric field at the center drops with the discharge power as shown in Figure 4, which depicts EM waves at different discharge powers. Thus, the decrease in the electric field can reduce the electron heating, leading to the T_e_ quenching.

**Figure 5:**
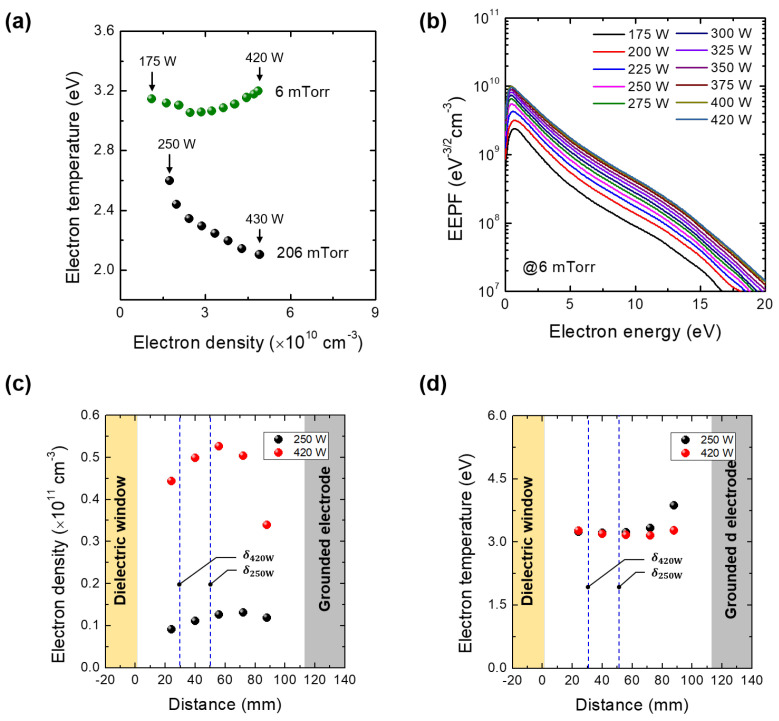
(**a**) Measured electron temperature (T${}_{e}$) over the measured electron densities with various discharge powers at 6 mTorr and 206 mTorr: at 6 mTorr, discharge power was varied from 175 to 420 W with 25 W increment and at 206 mTorr, from 250 W to 430 W with 25 W increment. (**b**) Electron energy probability function (EEPF) with various discharge powers at 6 mTorr. The Langmuir probe position is 58 mm, which is the center between the dielectric window (Al_2_O_3_) and the grounded electrode. (**c**) Electron density and (**d**) temperature profiles with discharge powers of 175 W and 420 W at 6 mTorr. The average skin depths ($\delta_{175W}$ of 50.7 ± 3.34 mm and $\delta_{420W}$ of 25.5 ± 2.22 mm) are marked as the dashed lines.

To prove the skin effect further, we measured the T_e_ in the low pressure regime. As shown in Figure 5a,b, the T_e_ quenching at 6 mTorr weakens. At low pressure, the skin depth is smaller than that at high pressure due to rare e-n collision events; the average skin depths with $n_{e}$ and T${}_{e}$, shown in Figure 5c,d, are 50.7 ± 3.34 mm and 25.5 ± 2.22 mm at 175 W and 420 W, respectively. The electric field is confined below the measurement position. Hence, electron heating is not effective at this position and the T${}_{e}$ slightly decreases with discharge power. On the other hand, electron density can rise since some electrons heated by the confined electric field cause ionization in the entire chamber as shown in Figure 5b. In addition, the slight increase of the T${}_{e}$ would result from the e-e collisions, that is, Maxwellization [19].

Aside from the skin depth, the EEPF reveals the electric field confinement. At this pressure, the $\lambda_{E}$ (≈200 mm) is much larger than the chamber scale, and the electron kinetics belong to non-local kinetics where electron heating is influenced not by the local electric field but rather by the average electric field via the entire chamber. In addition to the $\lambda_{E}$ scaling, the density profile shown in Figure 5b supports the non-local kinetics. The increase in the discharge power decreases the average electric field at the center since the δ is below the center as shown in Figure 5c. Furthermore, the decrease in the average field is lower than that of the local field drop due to the average effect. Thus, the electron heating slightly decreases with increasing discharge power and, thus, leads to small T${}_{e}$ quenching.

Hence, results measured at both low and high pressure indicate that the skin effect of EM waves dominates the T${}_{e}$ quenching mechanism.

## 4. Conclusions

In this work, we investigated the T${}_{e}$ quenching in an inductively coupled plasma source. We measured the T${}_{e}$ with a Langmuir probe at various discharge powers under 206 mTorr and 6 mTorr conditions. We found a large amount of T${}_{e}$ quenching about 0.5 eV at 206 mTorr, which is so large that it cannot be explained with the conventional scheme of multi-step ionization; the multi-step ionization effect on the decrease in T${}_{e}$ is lower than 0.1 eV. We analyzed the T${}_{e}$ quenching with the skin effect of the EM wave produced by the one-turn antenna. To estimate the average skin depth, we measured the $n_{e}$ and T${}_{e}$ distributions along the axial direction. Results show that the average skin depth decreases with increasing discharge power from 108.9 ± 14.6 mm at 250 W to 60.6 ± 8.4 mm at 430 W at 206 mTorr; the 60.6 mm is close to the chamber center where the Langmuir probe is located. The decrease in skin depth implies the decrease of electric field at the center. As this pressure belongs to the local kinetic regime, the decrease in electric field leads to the reduced electron heating and thus, quenching of the T${}_{e}$. Furthermore, we found a slight decrease of the T${}_{e}$ quenching at 6 mTorr in which the pressure regime belongs to non-local kinetics. The measured skin depth decreases from 50.7 ± 3.34 mm at 250 W to 25.5 ± 2.22 mm at 420 W. It results from the electric field confinement and non-local kinetic characteristics; the decrease in the averaged electric field is lower than the local electric field.

## Data Availability

The data presented in this study are available on request from the corresponding author.

## Appendix A

The electron–neutral (e-n) elastic collision mean free path ($\lambda_{\mathrm{el}}$) is defined as [5]

(A1) $$\lambda_{\mathrm{el}}=\frac{1}{n_{g}\sigma_{\mathrm{el}}},$$

where $n_{g}$ is gas density and $\sigma_{\mathrm{el}}$ is elastic collision cross section. The gas density is derived from the ideal gas law as

(A2) $$n_{g}=\frac{p}{k_{B}T_{g}},$$

where *p* is chamber pressure, $k_{B}$ is the Boltzmann constant, and $T_{g}$ is the gas temperature.

The electron–electron (e-e) collision frequency ($\nu_{\mathrm{ee}}$) is defined as [8]

(A3) $$\nu_{\mathrm{ee}}=2.91\times 10^{-6}n_{e}{T_{e}}^{-\frac{3}{2}}\ln\Lambda ,$$

where $n_{e}$ is electron density and lnΛ is defined as

(A4) $$\ln\Lambda =23-\ln\left(n_{e}^{\frac{1}{2}}T_{e}^{-\frac{3}{2}}\right).$$

The e-n collision frequency ($\nu_{m}$) is defined as

(A5) $$\nu_{m}=\frac{1}{n_{g}K_{\mathrm{el}}\left(T_{e}\right)}$$

$K_{\mathrm{el}}\left(T_{e}\right)$ is the rate constant for elastic collision with argon gas. The rate constant is given in [27] as

(A6) $$K_{\mathrm{el}}\left(T_{e}\right)=3.9\times 10^{-13}\exp\left(-\frac{4.6}{T_{e}}\right),$$

where T${}_{e}$ is electron temperature.

The excitation collision frequency ($\nu_{\mathrm{exc}}$) is defined as

(A7) $$\nu_{\mathrm{exc}}=\frac{1}{n_{g}K_{\mathrm{exc}}\left(T_{e}\right)}$$

$K_{\mathrm{exc}}\left(T_{e}\right)$ is the rate constant for excitation collision with argon gas defined as [5]

(A8) $$K_{\mathrm{exc}}\left(T_{e}\right)=2.48\times 10^{-14}T_{e}^{0.33}\exp\left(-\frac{12.78}{T_{e}}\right).$$

The ionization collision frequency ($\nu_{\mathrm{iz}}$) is defined as

(A9) $$\nu_{\mathrm{iz}}=\frac{1}{n_{g}K_{\mathrm{iz}}\left(T_{e}\right)}$$

$K_{\mathrm{iz}}\left(T_{e}\right)$ is the rate constant for ionization collision with argon gas defined as [5]

(A10) $$K_{\mathrm{iz}}\left(T_{e}\right)=2.34\times 10^{-14}T_{e}^{0.59}\exp\left(-\frac{17.44}{T_{e}}\right).$$

The excitation threshold energy ($E_{\mathrm{exc}}$) is 11.55 eV and the ionization threshold energy ($E_{\mathrm{iz}}$) is 15.76 eV [23,28].
